# Earthing: Health Implications of Reconnecting the Human Body to the Earth's Surface Electrons

**DOI:** 10.1155/2012/291541

**Published:** 2012-01-12

**Authors:** Gaétan Chevalier, Stephen T. Sinatra, James L. Oschman, Karol Sokal, Pawel Sokal

**Affiliations:** ^1^Developmental and Cell Biology Department, University of California at Irvine, Irvine, CA 92697, USA; ^2^Earth FX Inc., Palm Springs, CA 92262, USA; ^3^University of CT School of Medicine, c/o Optimum Health Building, 257 East Center Street, Farmington, CT 06040, USA; ^4^Nature's Own Research Association, Dover, NH 03821, USA; ^5^Department of Ambulatory Cardiology, Military Clinical Hospital, 85-681 Bydgoszcz, Poland; ^6^Department of Neurosurgery, Military Clinical Hospital, 85-681 Bydgoszcz, Poland

## Abstract

Environmental medicine generally addresses environmental factors with a negative impact on human health. However, emerging scientific research has revealed a surprisingly positive and overlooked environmental factor on health: direct physical contact with the vast supply of electrons on the surface of the Earth. Modern lifestyle separates humans from such contact. The research suggests that this disconnect may be a major contributor to physiological dysfunction and unwellness. Reconnection with the Earth's electrons has been found to promote intriguing physiological changes and subjective reports of well-being. Earthing (or grounding) refers to the discovery of benefits—including better sleep and reduced pain—from walking barefoot outside or sitting, working, or sleeping indoors connected to conductive systems that transfer the Earth's electrons from the ground into the body. This paper reviews the earthing research and the potential of earthing as a simple and easily accessed global modality of significant clinical importance.

## 1. Introduction

Environmental medicine focuses on interactions between human health and the environment, including factors such as compromised air and water and toxic chemicals, and how they cause or mediate disease. Omnipresent throughout the environment is a surprisingly beneficial, yet overlooked global resource for health maintenance, disease prevention, and clinical therapy: the surface of the Earth itself. It is an established, though not widely appreciated fact, that the Earth's surface possesses a limitless and continuously renewed supply of free or mobile electrons. The surface of the planet is electrically conductive (except in limited ultradry areas such as deserts), and its negative potential is maintained (i.e., its electron supply replenished) by the global atmospheric electrical circuit [1, 2]. 

Mounting evidence suggests that the Earth's negative potential can create a stable internal bioelectrical environment for the normal functioning of all body systems. Moreover, oscillations of the intensity of the Earth's potential may be important for setting the biological clocks regulating diurnal body rhythms, such as cortisol secretion [3].

It is also well established that electrons from antioxidant molecules neutralize reactive oxygen species (ROS, or in popular terms, free radicals) involved in the body's immune and inflammatory responses. The National Library of Medicine's online resource PubMed lists 7021 studies and 522 review articles from a search of “antioxidant + electron + free radical” [3]. It is assumed that the influx of free electrons absorbed into the body through direct contact with the Earth likely neutralize ROS and thereby reduce acute and chronic inflammation [4]. Throughout history, humans mostly walked barefoot or with footwear made of animal skins. They slept on the ground or on skins. Through direct contact or through perspiration-moistened animal skins used as footwear or sleeping mats, the ground's abundant free electrons were able to enter the body, which is electrically conductive [5]. Through this mechanism, every part of the body could equilibrate with the electrical potential of the Earth, thereby stabilizing the electrical environment of all organs, tissues, and cells.

Modern lifestyle has increasingly separated humans from the primordial flow of Earth's electrons. For example, since the 1960s, we have increasingly worn insulating rubber or plastic soled shoes, instead of the traditional leather fashioned from hides. Rossi has lamented that the use of insulating materials in post-World War II shoes has separated us from the Earth's energy field [6]. Obviously, we no longer sleep on the ground as we did in times past.

During recent decades, chronic illness, immune disorders, and inflammatory diseases have increased dramatically, and some researchers have cited environmental factors as the cause [7]. However, the possibility of modern disconnection with the Earth's surface as a cause has not been considered. Much of the research reviewed in this paper points in that direction.

In the late 19th century, a back-to-nature movement in Germany claimed many health benefits from being barefoot outdoors, even in cold weather [8]. In the 1920s, White, a medical doctor, investigated the practice of sleeping grounded after being informed by some individuals that they could not sleep properly “unless they were on the ground or connected to the ground in some way,” such as with copper wires attached to grounded-to-Earth water, gas, or radiator pipes. He reported improved sleeping using these techniques [9]. However, these ideas never caught on in mainstream society.

At the end of the last century, experiments initiated independently by Ober in the USA [10] and K. Sokal and P. Sokal [11] in Poland revealed distinct physiological and health benefits with the use of conductive bed pads, mats, EKG- and TENS-type electrode patches, and plates connected indoors to the Earth outside. Ober, a retired cable television executive, found a similarity between the human body (a bioelectrical, signal-transmitting organism) and the cable used to transmit cable television signals. When cables are “grounded” to the Earth, interference is virtually eliminated from the signal. Furthermore, all electrical systems are stabilized by grounding them to the Earth. K. Sokal and P. Sokal, meanwhile, discovered that grounding the human body represents a “universal regulating factor in Nature” that strongly influences bioelectrical, bioenergetic, and biochemical processes and appears to offer a significant modulating effect on chronic illnesses encountered daily in their clinical practices.

Earthing (also known as grounding) refers to contact with the Earth's surface electrons by walking barefoot outside or sitting, working, or sleeping indoors connected to conductive systems, some of them patented, that transfer the energy from the ground into the body. Emerging scientific research supports the concept that the Earth's electrons induce multiple physiological changes of clinical significance, including reduced pain, better sleep, a shift from sympathetic to parasympathetic tone in the autonomic nervous system (ANS), and a blood-thinning effect. The research, along with many anecdotal reports, is presented in a new book entitled *Earthing *[12].

## 2. Review of Earthing Papers

The studies summarized below involve indoor-testing methods under controlled conditions that simulate being barefoot outdoors.

### 2.1. Sleep and Chronic Pain

In a blinded pilot study, Ober recruited 60 subjects (22 males and 28 females) who suffered from self-described sleep disturbances and chronic muscle and joint pain for at least six months [10]. Subjects were randomly divided for the month-long study in which both groups slept on conductive carbon fiber mattress pads provided by Ober. Half the pads were connected to a dedicated Earth ground outside each subject's bedroom window, while the other half were “sham” grounded—not connected to the Earth. Results are presented in Table 1.

Most grounded subjects described symptomatic improvement while most in the control group did not. Some subjects reported significant relief from asthmatic and respiratory conditions, rheumatoid arthritis, PMS, sleep apnea, and hypertension while sleeping grounded. These results indicated that the effects of earthing go beyond reduction of pain and improvements in sleep.

### 2.2. Sleep, Stress, Pain, and Cortisol

A pilot study evaluated diurnal rhythms in cortisol correlated with changes in sleep, pain, and stress (anxiety, depression, and irritability), as monitored by subjective reporting [13]. Twelve subjects with complaints of sleep dysfunction, pain, and stress were grounded to Earth during sleep in their own beds using a conductive mattress pad for 8 weeks.

**Figure 1:**
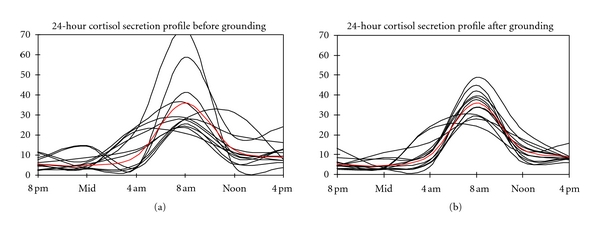
Cortisol levels before and after grounding. In unstressed individuals, the normal 24-hour cortisol secretion profile follows a predictable pattern: lowest around midnight and highest around 8 a.m. Graph (a) illustrates the wide variation of patterns among study participants prior to grounding, while (b) shows a realignment and normalization trend of patterns after six weeks of sleeping grounded.

In order to obtain a baseline measurement of cortisol, subjects chewed Dacron salvettes for 2 minutes and then placed them in time-labeled sampling tubes that were stored in a refrigerator. Self-administered sample collections began at 8 AM and were repeated every 4 hours. After 6 weeks of being grounded, subjects repeated this 24-hour saliva test. The samples were processed using a standard radioimmunoassay. A composite of the results is shown in Figure 1.

Subjective symptoms of sleep dysfunction, pain, and stress were reported daily throughout the 8-week test period. The majority of subjects with high- to out-of-range nighttime secretion levels experienced improvements by sleeping grounded. This is demonstrated by the restoration of normal day-night cortisol secretion profiles.

Eleven of 12 participants reported falling asleep more quickly, and all 12 reported waking up fewer times at night. Grounding the body at night during sleep also appears to positively affect morning fatigue levels, daytime energy, and nighttime pain levels.

About 30 percent of the general American adult population complain of sleep disruption, while approximately 10 percent have associated symptoms of daytime functional impairment consistent with the diagnosis of insomnia. Insomnia often correlates with major depression, generalized anxiety, substance abuse, dementia, and a variety of pain and physical problems. The direct and indirect costs of chronic insomnia have been estimated at tens of billions of dollars annually in the USA alone [14]. In view of the burdens of personal discomfort and health care costs, grounding the body during sleep seems to have much to offer. 

### 2.3. Earthing Reduces Electric Fields Induced on the Body

Voltage induced on a human body from the electrical environment was measured using a high-impedance measurement head. Applewhite, an electrical engineer and expert in the design of electrostatic discharge systems in the electronic industry, was both subject and author of the study [15]. Measurements were taken while ungrounded and then grounded using a conductive patch and conductive bed pad. The author measured the induced fields at three positions: left breast, abdomen, and left thigh.

**Figure 2:**
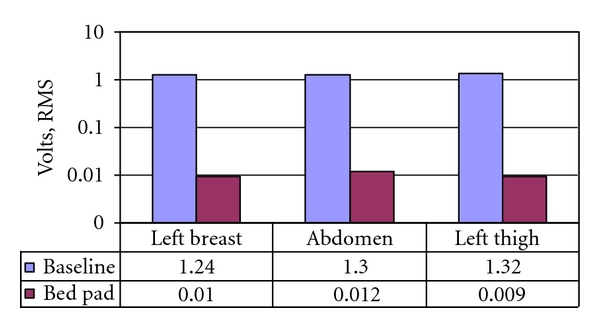
Effect of bed pad grounding on 60 Hz mode.

Each method (patch and sheet) immediately reduced the common alternating current (AC) 60 Hz ambient voltage induced on the body by a highly significant factor of about 70 on average.  Figure 2 shows this effect.

The study showed that when the body is grounded, its electrical potential becomes equalized with the Earth's electrical potential through a transfer of electrons from the Earth to the body. This, in turn, prevents the 60 Hz mode from producing an AC electric potential at the surface of the body and from producing perturbations of the electric charges of the molecules inside the body. The study confirms the “umbrella” effect of earthing the body explained by Nobel Prize winner Richard Feynman in his lectures on electromagnetism [16]. Feynman said that when the body potential is the same as the Earth's electric potential (and thus grounded), it becomes an extension of the Earth's gigantic electric system. The Earth's potential thus becomes the “working agent that cancels, reduces, or pushes away electric fields from the body.”

Applewhite was able to document changes in the ambient voltage induced on the body by monitoring the voltage drop across a resistor. This effect clearly showed the “umbrella effect” described above. The body of the grounded person is not subject to the perturbation of electrons and electrical systems.

Jamieson asks whether the failure to appropriately ground humans is a factor contributing to the potential consequences of electropollution in office settings [17]. Considerable debate exists on whether electromagnetic fields in our environment cause a risk to health [18], but there is no question that the body reacts to the presence of environmental electric fields. This study demonstrates that grounding essentially eliminates the ambient voltage induced on the body from common electricity power sources.

### 2.4. Physiological and Electrophysiological Effects

#### 2.4.1. Reductions in Overall Stress Levels and Tension and Shift in ANS Balance

Fifty-eight healthy adult subjects (including 30 controls) participated in a randomized double-blind pilot study investigating earthing effects on human physiology [19]. Earthing was accomplished with a conductive adhesive patch placed on the sole of each foot. A biofeedback system recorded electrophysiological and physiological parameters. Experimental subjects were exposed to 28 minutes in the unearthed condition followed by 28 minutes with the earthing wire connected. Controls were unearthed for 56 minutes.

Upon earthing, about half the subjects showed an abrupt, almost instantaneous change in root mean square (rms) values of electroencephalograms (EEGs) from the left hemisphere (but not the right hemisphere) at all frequencies analyzed by the biofeedback system (beta, alpha, theta, and delta).

All grounded subjects presented an abrupt change in rms values of surface electromyograms (SEMGs) from right and left upper trapezius muscles. Earthing decreased blood volume pulse (BVP) in 19 of 22 experimental subjects (statistically significant) and in 8 of 30 controls (not significant). Earthing the human body showed significant effects on electrophysiological properties of the brain and musculature, on the BVP, and on the noise and stability of electrophysiological recordings. Taken together, the changes in EEG, EMG, and BVP suggest reductions in overall stress levels and tensions and a shift in ANS balance upon earthing. The results extend the conclusions of previous studies.

#### 2.4.2. Confirming Shift from Sympathetic to Parasympathetic Activation

A multiparameter double-blind study was designed to reproduce and expand on previous electrophysiological and physiological parameters measured immediately after grounding with an improved methodology and state-of-the-art equipment [20]. Fourteen men and 14 women, in good health, ages 18–80, were tested while seated in a comfortable recliner during 2-hour grounding sessions, leaving time for signals to stabilize before, during, and after grounding (40 minutes for each period). Sham 2-hour grounding sessions were also recorded with the same subjects as controls. For each session, statistical analyses were performed on four 10-minute segments: before and after grounding (sham grounding for control sessions) and before and after ungrounding (sham ungrounding for control sessions). The following results were documented:

- an immediate decrease (within a few seconds) in skin conductance (SC) at grounding and an immediate increase at ungrounding. No change was seen for the control (sham grounding) sessions;
- respiratory rate (RR) increased during grounding, an effect that lasted after ungrounding. RR variance increased immediately after grounding and then decreased;
- blood oxygenation (BO) variance decreased during grounding, followed by a dramatic increase after ungrounding;
- pulse rate (PR) and perfusion index (PI) variances increased toward the end of the grounding period, and this change persisted after ungrounding.

The immediate decrease in SC indicates a rapid activation of the parasympathetic nervous system and corresponding deactivation of the sympathetic nervous system. The immediate increase in SC at cessation of grounding indicates an opposite effect. Increased RR, stabilization of BO, and slight rise in heart rate suggest the start of a metabolic healing response necessitating an increase in oxygen consumption.

#### 2.4.3. Immune Cell and Pain Responses with Delayed-Onset Muscle Soreness Induction

Pain reduction from sleeping grounded has been documented in previous studies [10, 13]. This pilot study looked for blood markers that might differentiate between grounded and ungrounded subjects who completed a single session of intense, eccentric exercise resulting in delayed-onset muscle soreness (DOMS) of the gastrocnemius [21]. If markers were able to differentiate these groups, future studies could be done in greater detail with a larger subject base. DOMS is a common complaint in the fitness and athletic world following excessive physical activity and involves acute inflammation in overtaxed muscles. It develops in 14 to 48 hours and persists for more than 96 hours [22]. No known treatment reduces the recovery period, but apparently massage and hydrotherapy [23–25] and acupuncture [26] can reduce pain.

Eight healthy men ages 20–23 were put through a similar routine of toe raises while carrying on their shoulders a barbell equal to one-third of their body weight. Each participant was exercised individually on a Monday morning and then monitored for the rest of the week while following a similar eating, sleeping, and living schedule in a hotel. The group was randomly divided in half and either grounded or sham grounded with the use of a conductive patch placed at the sole of each foot during active hours and a conductive sheet at night. Complete blood counts, blood chemistry, enzyme chemistry, serum and saliva cortisol, magnetic resonance imaging and spectroscopy, and pain levels (a total of 48 parameters) were taken at the same time of day before the eccentric exercise and at 24, 48, and 72 hours afterwards. Parameters consistently differing by 10 percent or more, normalized to baseline, were considered worthy of further study.

Parameters that differed by these criteria included white blood cell counts, bilirubin, creatine kinase, phosphocreatine/inorganic phosphate ratios, glycerolphosphorylcholine, phosphorylcholine, the visual analogue pain scale, and pressure measurements on the right gastrocnemius.

**Figure 3:**
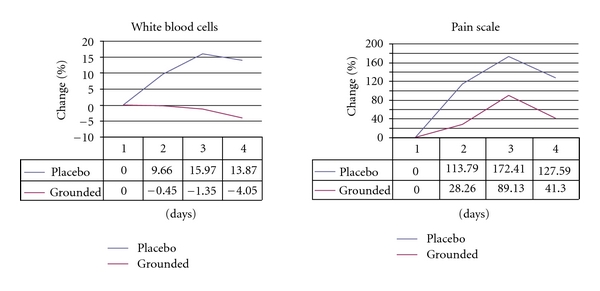
Delayed onset muscle soreness and grounding. Consistent with all measurements, ungrounded subjects expressed the perception of greater pain. Related to the pain finding was evidence of a muted white blood cell response indicating that a grounded body experiences less inflammation.

The results showed that grounding the body to the Earth alters measures of immune system activity and pain. Among the ungrounded men, for instance, there was an expected, sharp increase in white blood cells at the stage when DOMS is known to reach its peak and greater perception of pain (see Figure 3). This effect demonstrates a typical inflammatory response. In comparison, the grounded men had only a slight decrease in white blood cells, indicating scant inflammation, and, for the first time ever observed, a shorter recovery time. Brown later commented that there were “significant differences” in the pain these men reported [12]. 

#### 2.4.4. Heart Rate Variability

The rapid change in skin conductance reported in an earlier study led to the hypothesis that grounding may also improve heart rate variability (HRV), a measurement of the heart's response to ANS regulation. A double-blind study was designed with 27 participants [27]. Subjects sat in a comfortable reclining chair. Four transcutaneous electrical nerve stimulation (TENS) type adhesive electrode patches were placed on the sole of each foot and on each palm.

Participants served as their own controls. Each participant's data from a 2-hour session (40 minutes of which was grounded) were compared with another 2-hour sham-grounded session. The sequence of grounding versus sham-grounding sessions was assigned randomly.

During the grounded sessions, participants had statistically significant improvements in HRV that went way beyond basic relaxation results (which were shown by the nongrounded sessions). Since improved HRV is a significant positive indicator on cardiovascular status, it is suggested that simple grounding techniques be utilized as a basic integrative strategy in supporting the cardiovascular system, especially under situations of heightened autonomic tone when the sympathetic nervous system is more activated than the parasympathetic nervous system.

#### 2.4.5. Reduction of Primary Indicators of Osteoporosis, Improvement of Glucose Regulation, and Immune Response

K. Sokal and P. Sokal, cardiologist and neurosurgeon father and son on the medical staff of a military clinic in Poland, conducted a series of experiments to determine whether contact with the Earth via a copper conductor can affect physiological processes [11]. Their investigations were prompted by the question as to whether the natural electric charge on the surface of the Earth influences the regulation of human physiological processes.

Double-blind experiments were conducted on groups ranging from 12 to 84 subjects who followed similar physical activity, diet, and fluid intake during the trial periods. Grounding was achieved with a copper plate (30 mm × 80 mm) placed on the lower part of the leg, attached with a strip so that it would not come off during the night. The plate was connected by a conductive wire to a larger plate (60 mm × 250 mm) placed in contact with the Earth outside.

In one experiment with nonmedicated subjects, grounding during a single night of sleep resulted in statistically significant changes in concentrations of minerals and electrolytes in the blood serum: iron, ionized calcium, inorganic phosphorus, sodium, potassium, and magnesium. Renal excretion of both calcium and phosphorus was reduced significantly. The observed reductions in blood and urinary calcium and phosphorus directly relate to osteoporosis. The results suggest that Earthing for a single night reduces primary indicators of osteoporosis.

Earthing continually during rest and physical activity over a 72-hour period decreased fasting glucose among patients with non-insulin-dependent diabetes mellitus. Patients had been well controlled with glibenclamide, an antidiabetic drug, for about 6 months, but at the time of study had unsatisfactory glycemic control despite dietary and exercise advice and glibenclamide doses of 10 mg/day.

K. Sokal and P. Sokal drew blood samples from 6 male and 6 female adults with no history of thyroid disease. A single night of grounding produced a significant decrease of free tri-iodothyronine and an increase of free thyroxin and thyroid-stimulating hormone. The meaning of these results is unclear but suggests an earthing influence on hepatic, hypothalamus, and pituitary relationships with thyroid function. Ober et al. [12] have observed that many individuals on thyroid medication reported symptoms of hyperthyroid, such as heart palpitations, after starting grounding. Such symptoms typically vanish after medication is adjusted downward under medical supervision. Through a series of feedback regulations, thyroid hormones affect almost every physiological process in the body, including growth and development, metabolism, body temperature, and heart rate. Clearly, further study of earthing effects on thyroid function is needed.

In another experiment, the effect of grounding on the classic immune response following vaccination was examined. Earthing accelerated the immune response, as demonstrated by increases in gamma globulin concentration. This result confirms an association between earthing and the immune response, as was suggested in the DOMS study [21].

K. Sokal and P. Sokal conclude that earthing the human body influences human physiological processes, including increasing the activity of catabolic processes and may be “the primary factor regulating endocrine and nervous systems.”

#### 2.4.6. Altered Blood Electrodynamics

Since grounding produces changes in many electrical properties of the body [1, 15, 19, 28], a next logical step was to evaluate the electrical property of the blood. A suitable measure is the zeta potential of red blood cells (RBCs) and RBC aggregation. Zeta potential is a parameter closely related to the number of negative charges on the surface of an RBC. The higher the number, the greater the ability of the RBC to repel other RBCs. Thus, the greater the zeta potential the less coagulable is the blood.

Ten relatively healthy subjects participated in the study [29]. They were seated comfortably in a reclining chair and were grounded for two hours with electrode patches placed on their feet and hands, as in previous studies. Blood samples were taken before and after. 

Grounding the body to the earth substantially increases the zeta potential and decreases RBC aggregation, thereby reducing blood viscosity. Subjects in pain reported reduction to the point that it was almost unnoticeable. The results strongly suggest that earthing is a natural solution for patients with excessive blood viscosity, an option of great interest not just for cardiologists, but also for any physician concerned about the relationship of blood viscosity, clotting, and inflammation. In 2008, Adak and colleagues reported the presence of both hypercoagulable blood and poor RBC zeta potential among diabetics. Zeta potential was particularly poor among diabetics with cardiovascular disease [30].

## 3. Discussion

Until now, the physiological significance and possible health effects of stabilizing the internal bioelectrical environment of an organism have not been a significant topic of research. Some aspects of this, however, are relatively obvious. In the absence of Earth contact, internal charge distribution will not be uniform, but instead will be subject to a variety of electrical perturbations in the environment. It is well known that many important regulations and physiological processes involve events taking place on cell and tissue surfaces. In the absence of a common reference point, or “ground,” electrical gradients, due to uneven charge distribution, can build up along tissue surfaces and cell membranes.

We can predict that such charge differentials will influence biochemical and physiological processes. First, the structure and functioning of many enzymes are sensitive to local environmental conditions. Each enzyme has an optimal pH that favors maximal activity. A change in the electrical environment can alter the pH of biological fluids and the charge distribution on molecules and thereby affect reaction rates. The pH effect results because of critical charged amino acids at the active site of the enzyme that participate in substrate binding and catalysis. In addition, the ability of a substrate or enzyme to donate or accept hydrogen ions is influenced by pH.

Another example is provided by voltage-gated ion channels, which play critical biophysical roles in excitable cells such as neurons. Local alterations in the charge profiles around these channels can lead to electrical instability of the cell membrane and to the inappropriate spontaneous activity observed during certain pathological states [31].

Earthing research offers insights into the clinical potential of barefoot contact with the Earth, or simulated barefoot contact indoors via simple conductive systems, on the stability of internal bioelectrical function and human physiology. Initial experiments resulted in subjective reports of improved sleep and reduced pain [10]. Subsequent research showed that improved sleep was correlated with a normalization of the cortisol day-night profile [13]. The results are significant in light of the extensive research showing that lack of sleep stresses the body and contributes to many detrimental health consequences. Lack of sleep is often the result of pain. Hence, reduction of pain might be one reason for the benefits just described.

Pain reduction from sleeping grounded has been confirmed in a controlled study on DOMS. Earthing is the first intervention known to speed recovery from DOMS [21]. Painful conditions are often the result of various kinds of acute or chronic inflammation conditions caused in part by ROS generated by normal metabolism and also by the immune system as part of the response to injury or trauma. Inflammation can cause pain and loss of range of motion in joints. Inflammatory swelling can put pressure on pain receptors (nocireceptors) and can compromise the microcirculation, leading to ischemic pain. Inflammation can cause the release of toxic molecules that also activate pain receptors. Modern biomedical research has also documented a close relationship between chronic inflammation and virtually all chronic diseases, including the diseases of aging, and the aging process itself. The steep rise in inflammatory diseases, in fact, has been recently called “inflamm-aging” to describe a progressive inflammatory status and a loss of stress-coping ability as major components of the aging process [32]. 

Reduction in inflammation as a result of earthing has been documented with infrared medical imaging [28] and with measurements of blood chemistry and white blood cell counts [21]. The logical explanation for the anti-inflammatory effects is that grounding the body allows negatively charged antioxidant electrons from the Earth to enter the body and neutralize positively charged free radicals at sites of inflammation [28]. Flow of electrons from the Earth to the body has been documented [15].

A pilot study on the electrodynamics of red blood cells (zeta potential) has revealed that earthing significantly reduces blood viscosity, an important but neglected parameter in cardiovascular diseases and diabetes [29], and circulation in general. Thus, thinning the blood may allow for more oxygen delivery to tissues and further support the reduction of inflammation.

Stress reduction has been confirmed with various measures showing rapid shifts in the ANS from sympathetic to parasympathetic dominance, improvement in heart rate variability, and normalization of muscle tension [19, 20, 27]. 

Not reported here are many observations over more than two decades by Ober et al. [12] and K. Sokal and P. Sokal [11] indicating that regular earthing may improve blood pressure, cardiovascular arrhythmias, and autoimmune conditions such as lupus, multiple sclerosis, and rheumatoid arthritis. Some effects of earthing on medication are described by Ober et al. [12] and at the website: http://www.earthinginstitute.net/. As an example, the combination of earthing and coumadin has the potential to exert a compounded blood thinning effect and must be supervised by a physician. Multiple anecdotes of elevated INR have been reported. INR (international normalized ratio) is a widely used measurement of coagulation. The influence of earthing on thyroid function and medication has been described earlier.

From a practical standpoint, clinicians could recommend outdoor “barefoot sessions” to patients, weather, and conditions permitting. Ober et al. [12] have observed that going barefoot as little as 30 or 40 minutes daily can significantly reduce pain and stress, and the studies summarized here explain why this is the case. Obviously, there is no cost for barefoot grounding. However, the use of conductive systems while sleeping, working, or relaxing indoors offer a more convenient and routine-friendly approach.

## 4. Conclusion

De Flora et al. wrote the following: “Since the late 20th century, chronic degenerative diseases have overcome infectious disease as the major causes of death in the 21st century, so an increase in human longevity will depend on finding an intervention that inhibits the development of these diseases and slows their progress” [33].

Could such an intervention be located right beneath our feet? Earthing research, observations, and related theories raise an intriguing possibility about the Earth's surface electrons as an untapped health resource—the Earth as a “global treatment table.” Emerging evidence shows that contact with the Earth—whether being outside barefoot or indoors connected to grounded conductive systems—may be a simple, natural, and yet profoundly effective environmental strategy against chronic stress, ANS dysfunction, inflammation, pain, poor sleep, disturbed HRV, hypercoagulable blood, and many common health disorders, including cardiovascular disease. The research done to date supports the concept that grounding or earthing the human body may be an essential element in the health equation along with sunshine, clean air and water, nutritious food, and physical activity.

## Figures and Tables

**Table 1:** Subjective sleep, pain, and well-being feedback.

Categories	Test subjects*		Control subjects**	
	Same	Improved	Same	Improved
Time to fall asleep	4 = 15%	23 = 85%	20 = 87%	3 = 13%
Quality of sleep	2 = 7%	25 = 93%	20 = 87%	3 = 13%
Wake feeling rested	0 = 0%	27 = 100%	20 = 87%	3 = 13%
Muscles stiffness and pain	5 = 18%	22 = 82%	23 = 100%	0 = 0%
Chronic back and/or joint pain	7 = 26%	20 = 74%	23 = 100%	0 = 0%
General well-being	6 = 22%	21 = 78%	20 = 87%	3 = 13%

*Reports not received from three participants.

**Reports not received from seven participants.
